# Unusual primary location of non-Hodgkin lymphoma in the uterine body with compressive form: About case

**DOI:** 10.1016/j.amsu.2021.102607

**Published:** 2021-07-27

**Authors:** F. El Miski, A. Hanafi, E. Telmoudi, Z. Bousada, I.El Abbassi, M. Jalal, A. Lamrissi, K. Fichtali, S. Bouhya

**Affiliations:** Obstetrics and Gynecology Department, Univesity Hospital Center Ibn Rochd, Casablanca, 20100, Morocco

**Keywords:** Non-hodgkin's lymphoma, Uterus, Diagnosis, Immunochemotherapy, Case report

## Abstract

**Introduction:**

Primary non-Hodgkin's malignant lymphoma (NHML) of the uterine body is an extremely rare localization since only eleven cases have been reported in the literature.

**Case presentation:**

We report a case of primary NHML of the uterine body discovered during a hysterectomy performed for a uterine mass. The primary character of NHML of the uterine body was retained in view of the absence of extra-genital localization in the clinical, biological and radiological workup (Ann Arbor stage IE) and there were no signs of recurrence during follow-up. The patient received anti-CD20 immunochemotherapy (rituximab-CHOP and rituximab-VCAP combinations) and at 12 months follow-up, she is in complete remission.

**Discussion:**

The diagnosis of primary and isolated NHML of the uterine body is based on a clinical and further examination and regular follow-up for several months. The treatment is not codified; surgery, poly-chemotherapy and radiotherapy are the different therapeutic modalities. Rituximab-CHOP immunochemotherapy is currently the reference treatment for primary malignant lymphomas of the uterine body particularly in young patients who wish to become pregnant. The prognosis depends mainly on two factors: age and Ann Arbor stage.

**Conclusion:**

Primary uterine lymphomas are rare tumors of unknown etiopathogeny and of non-specific clinical presentation, the role of the various treatments remains difficult to evaluate due to the small number of published cases.

## Highlights

• Primary non-Hodgkin's malignant lymphoma (NHML) of the uterine body is an exceptional localization
•Its rare tumors of unknown etiopathogeny and of non-specific clinical presentation
•The diagnosis is based on a rigorous clinical and paraclinical work-up and regular follow-up for several months
•The treatment is not codified; Rituximab-CHOP immunochemotherapy is currently the reference treatment.
•The prognosis depends mainly on two factors: age and Ann Arbor stage.

## 1 Introduction

Uterine non-Hodgkin's malignant lymphoma (NHML) is a rare tumor entity that can occur in two distinct diagnostic circumstances: most often in the context of a secondary uterine location of a disseminated lymphoma, rarely as a primary uterine lymphoma. Primary NHML of the uterine body is even more unusual than that of the cervix. These unusual tumors pose various diagnostic and therapeutic problems. We hope, through a case of primary non-Hodgkin's malignant lymphoma of the uterine body in the gynecology obstetrics department of the Ibn Rochd hospital of Casablanca, to contribute to the study of this rare localization of lymphoma. This work has been reported with respect to the SCARE 2020 criteria [1].

## 2 Case report

The 45-year-old patient, mother of 3 children, sexually active, had a history of bilateral tympanoplasty, cholecystectomy and recurrent spontaneous abortions. She consulted our service for minimal spontaneous metrorrhagia evolving for 8 months, secondarily complicated by oliguria. The gynecological examination revealed a homogeneous normal cervix without any mass on its surface with minimal reddish metrorrhagia from inside the uterus. The pelvic examination revealed an enlarged uterus, reaching two fingerbreadths above the pubic bone, with a peri-uterine corporal-isthmic mass engulfing the uterus without infiltration of the parameters or the vagina. The lymph node areas were are without palpable adenopathy and the rest of the general clinical examination was normal. Pelvic ultrasound revealed an enlarged uterus with the presence of a heterogeneous hypoechoic corporal-ischemic region mass measuring 70.6 × 47 mm that may be related to a large sub mucosal myoma.

**Fig. 1:**
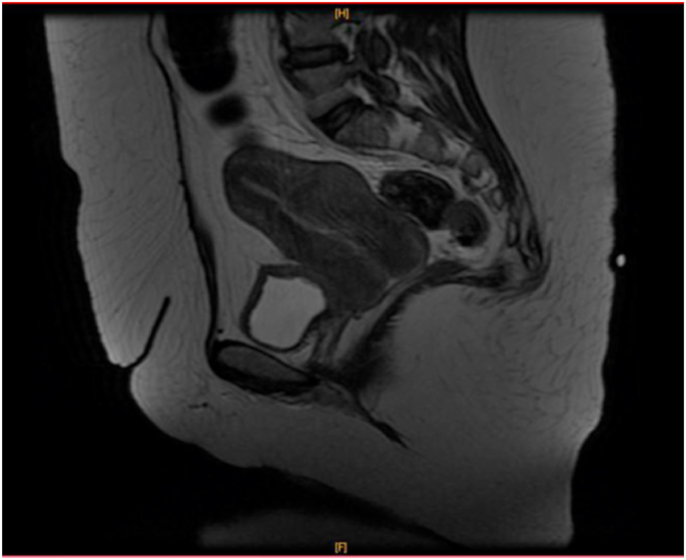
45-year-old patient, metrorrhagia with enlarged uterus on pelvic MRI - sagittal section (T2 sequence): Swollen appearance at cervical-corporeo-isthmic level with deformation of the contours and cavity line, measuring 7 × 5 cm with no pelvic lymphadenopathy. The injection of gadolinium had determined a frank and homogeneous enhancement of the uterine background and a more discreet enhancement in the thickening of the rest of the uterus. With no infiltration of the vagina, bladder, parameters, and dilatation of the two ureters more marked on the right.

Renal ultrasound had shown significant bilateral hydronephrosis predominantly on the right with normal cortical index. Pelvic magnetic resonance imaging (MRI) showed a swollen appearance at the cervical-corporeal-isthmic level with deformation of the contours and the cavity line, measuring 7× 5 cm that could be related to a cervical-corporeal-isthmic interstitial fibroid with the absence of pelvic adenopathies. The injection of iodinated contrast medium had determined a frank and homogeneous enhancement of the uterine fundus without infiltration of the vagina, the bladder and the parametrium. The examination also showed a dilatation of both ureters more marked on the right. [Fig. 1].

The urinary tract scan revealed a significant bilateral pyelocalic dilatation, with a diameter of the ureters measuring 18 mm on the right and 16 mm on the left, secondary to a pelvic obstacle represented by a uterine mass at the isthmic region. The biological workup showed renal failure secondary to a pelvic obstruction related to a corporal-isthmic mass with a creatinine concentration raised to 142.72 mg/l.

The patient had undergone 3 sessions of hemodialysis prior to the installation of the double J catheter on both sides, with a rehydration regimen that allowed for an improvement in renal function as well as normalization of creatinine levels. The patient benefited from a laparotomy with an enlarged uterus at the corporal-isthmic level of a hard homogeneous tumor mass of about 7 cm. The mass was inoperable and its biopsy was difficult despite its extent. This led the surgical team to perform a subtotal inter-annexal hysterectomy. The postoperative course was uncomplicated.

The anatomo-pathological study of the specimen revealed in the uterine body a cellular proliferation infiltrating the myometrium of lymphoid appearance without endometrial involvement. This lymphomatous proliferation made of large cells with ill-defined cytoplasm and irregular nuclei having a fine chromatin with one or two nucleoli of variable arrangement and frequent mitoses.

**Fig. 2:**
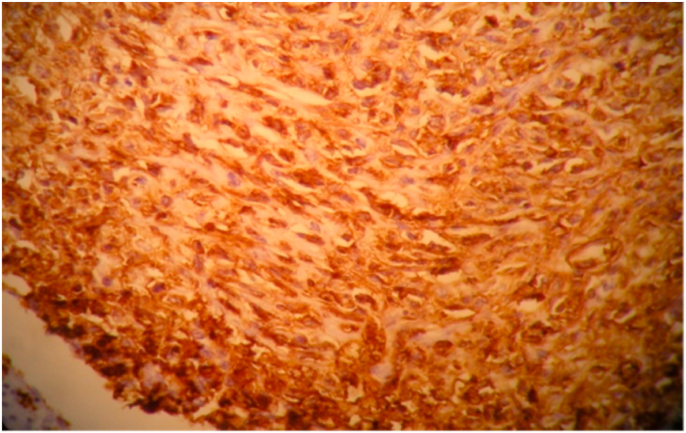
Diffuse lymphoma proliferation with large cells expressing diffuse and intense CD20 (immunohistochemistry).

**Fig. 3:**
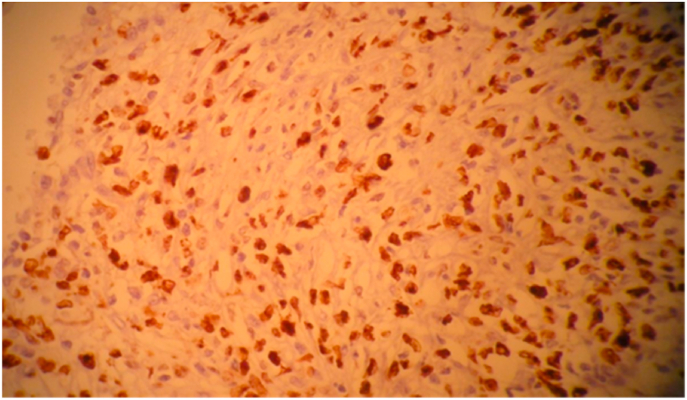
Immunolabeling with Ki67 showing a cycle rate of about 60%.

The immunohistochemistry study showed a diffuse expression of CD20 by the above described tumor cells, absence of expression of CD3 - CD5 - CD10 - CD30 - CD23 - BCL2 and cytokeratin AE1/AE3, a proliferation index evaluated by Ki 67 estimated at 60% of the above-described lymphomatous infiltration. The diagnosis of myometrial localization of a diffuse large B-cell malignant non-Hodgkin's lymphoma was therefore retained [Fig. 2, Fig. 3].

The extension workup including clinical examination, thoracic-abdominal-pelvic CT scan, and upper gastrointestinal endoscopy was normal; osteomedullary biopsy and hematologic workup were also normal. The primary character of the lymphoma was retained on the absence of other peripheral localizations with no lymphomatous infiltration at the osteomedullary biopsy.

The patient then received immunochemotherapy combining three cycles of rituximab-CHOP (adriamycin-cyclophosphamide-vincristine®-prednisone) and one cycle of rituximab-VCAP (vindesine-cyclophosphamide-adriamycin-prednisone) with good clinical and hematological tolerance. The patient was then followed regularly and showed no sign of disease recurrence at 12 months.

## 3 Discussion

The NHML is one of the hematologic malignancies that affects mainly lymphoid tissue organs. The genital area is an exclusively rare location, representing less than 1% of lymphomas [2].

The primary NHLM of the uterine body remains a controversial subject because of the number of so-called “primary” uterine lymphomas reported in the literature. Genital involvement in extensive non-Hodgkin's lymphoma refractory to chemotherapy is well known: 40% of cases in the autopsy series of Rosenberg et al. [3]. In contrast, the frequency of primary lymphoma of the uterine body is not well elucidated.

For most authors, the diagnosis of primary uterine lymphoma is based on the combination of the four criteria of Fox and More [4].

- disease confined to the uterus at the time of diagnosis;
- absence of dissemination from the primary site after treatment for at least several months;
- the absence of leukemia at the time of diagnosis;
- the follow-up of several months to be able to eliminate an extra genital recurrence.

Our observation is of twofold interest because, on the one hand, primary involvement of the uterine body remains exceptional compared to involvement of the cervix [5] and, on the other hand, the clinical symptomatology represented by the compressive form of the urinary tract.

The etiopathogenesis of the genesis of primary uterine lymphomas remains poorly elucidated. However, hypotheses have incriminated several factors: chronic inflammation, autoimmune nature or not, infectious agents such as Epstein-Barr virus (EBV) seems to play an important etiological role in the pathogenesis of extra-ganglionic lymphomas [6,7].

The disease affects all age groups indiscriminately with an average age of 52 years [7]. These rare tumors of the uterine body have no specific symptomatology and are most often revealed by peri- or post-menopausal metrorrhagia [8]. In our case, in addition to the metrorrhagia, the urinary compressive signs were reminiscent of the compressive form of lymphomas; this compressive character is not described in the literature.

The positive diagnosis is often made retrospectively on histological analysis of a hysterectomy specimen. Diffuse large-cell B-cell NHML is the most frequent histological type, whereas T-cell NHL is rarer. Immunohistochemical techniques can confirm the lymphoid nature and specify its B or T type. The most frequent macroscopic appearance is entophytic with a diffuse thickening of the body wall without associated mucosal abnormality [9].

The most widely used, most accurate, and most predictive classification of evolution and exact extension is the Ann Arbor classification used for extra-nodal NHML. Medical imaging has a fundamental role in the management of NHML of the uterine body, in particular: initial diagnosis, pre-therapeutic extension assessment and post-therapeutic monitoring during remissions and recurrences [9].

The advent of nuclear medicine has allowed an excellent evaluation of the extension, the workup often includes lymphangiography and/or gallium-67 scintigraphy and now positron emission tomography (TEP-scan). Cross-sectional imaging (ultrasound, CT and MRI) has profoundly modified the pre-therapeutic workup and is becoming the predominant and sometimes exclusive imaging before treatment. Ultrasound allows a good study of the abdomino-pelvic lymph node chains and thanks to its Doppler sequence it is possible to differentiate between tumoral and inflammatory lymph node as well as normal sized but pathological lymph nodes [10].

The superiority of CT scan is clear for obese patients, whereas for lean patients, ultrasound may be more effective. CT only detects pathological nodes when they have increased in size or number, and is unable, even after iodine injection, to differentiate inflammatory or reactive lymph nodes from invasive ones.

The MRI allows, contrary to CT, a better study of the iliac lymph nodes and the detection of pathological nodes of normal size [9]. Because of their rarity and the absence of randomized studies, NHML treatment is not codified. Surgery, polychemotherapy and radiotherapy are the different therapeutic modalities. In the literature, the combination of hysterectomy and chemotherapy gives encouraging results. However, the results of the combination of hysterectomy and radiotherapy are disappointing, with most patients dying in relapse [11,12].

Rituximab is a monoclonal antibody directed against the CD20 surface antigen (B phenotype). Rituximab-CHOP immunochemotherapy (6–8 courses) is currently the reference treatment for primary malignant lymphomas of the uterine body. This treatment makes it possible to avoid hysterectomy, particularly in young patients who wish to become pregnant.

The evolution of primary lymphoma of the uterine body differs according to the authors: some argue that it is a more negative localization than cervical and/or vaginal involvement; others report that body involvement does not worsen the prognosis.The prognosis depends mainly on two factors: age and Ann Arbor stage [12,14].

## 4 Conclusion

Primary uterine lymphomas are rare tumors of unknown etiopathogeny and of non-specific clinical presentation. The diagnosis of primary and isolated NHML of the uterine body is based on a rigorous clinical and paraclinical work-up and regular follow-up for several months. The role of the various treatments remains difficult to evaluate due to the small number of published cases. The therapeutic choice of the combination of primary surgery followed by anti-CD20 immunochemotherapy rituximab-CHOP and rituximab-VCAP combinations with a good evolution consolidate the results of the literature.

## Sources of funding

None.

## Ethical approval

I declare on my honor that the ethical approval has been exempted by my establishment.

## Consent

Written informed consent for publication of their clinical details and/or clinical images was obtained from the patient.

## Author contribution

El Miski Fatiha: Corresponding authorr writing the paper.

## Guarantor

El Miski Fatiha.

## Research registration number

None.

## Declaration of competing interest

The authors declare having no conflicts of interest for this article.
